# Alphaherpesvirus Major Tegument Protein VP22: Its Precise Function in the Viral Life Cycle

**DOI:** 10.3389/fmicb.2020.01908

**Published:** 2020-08-07

**Authors:** Liping Wu, Anchun Cheng, Mingshu Wang, Renyong Jia, Qiao Yang, Ying Wu, Dekang Zhu, Xinxin Zhao, Shun Chen, Mafeng Liu, Shaqiu Zhang, Xuming Ou, Sai Mao, Qun Gao, Di Sun, Xinjian Wen, Yunya Liu, Yanling Yu, Ling Zhang, Bin Tian, Leichang Pan, Xiaoyue Chen

**Affiliations:** ^1^Institute of Preventive Veterinary Medicine, Sichuan Agricultural University, Chengdu, China; ^2^Key Laboratory of Animal Disease and Human Health of Sichuan Province, Sichuan Agricultural University, Chengdu, China; ^3^Avian Disease Research Center, College of Veterinary Medicine, Sichuan Agricultural University, Chengdu, China

**Keywords:** alphaherpesvirus, tegument protein VP22, secondary envelopment, viral replication, immune evasion

## Abstract

Alphaherpesviruses are zoonotic pathogens that can cause a variety of diseases in humans and animals and severely damage health. Alphaherpesvirus infection is a slow and orderly process that can lie dormant for the lifetime of the host but may be reactivated when the immune system is compromised. All alphaherpesviruses feature a protein layer called the tegument that lies between the capsid and the envelope. Virus protein (VP) 22 is one of the most highly expressed tegument proteins; there are more than 2,000 copies of this protein in each viral particle. VP22 can interact with viral proteins, cellular proteins, and chromatin, and these interactions play important roles. This review summarizes the latest literature and discusses the roles of VP22 in viral gene transcription, protein synthesis, virion assembly, and viral cell-to-cell spread with the purpose of enhancing understanding of the life cycle of herpesviruses and other pathogens in host cells. The molecular interaction information herein provides important reference data.

## Introduction

The members of *Herpesviridae* are double-stranded DNA (dsDNA) viruses with a tegument structure (Crump, 2018). Currently, there are more than 120 known types of herpesviruses. In 1981, the International Commission on Taxonomy of Viruses (ICTV) recommended that the herpesvirus family be divided into three subfamilies (*Alphaherpesvirinae*, *Betaherpesvirinae*, and *Gammaherpesvirinae*), each subfamily of which has many unclassified viruses. Additionally, many herpesviruses have not yet been classified into a specific subfamily (Roizman et al., 1981). The herpes simplex virus types 1 and 2 (HSV-1 and HSV-2, respectively), duck enteritis virus (DEV), varicella-zoster virus (VZV), bovine herpesvirus 1 (BoHV-1), Marek’s disease virus (MDV), and pseudorabies virus (PRV) all belong to the *Alphaherpesvinae* subfamily. Human and murine cytomegalovirus (HCMV and MCMV, respectively) belong to the *Betaherpesvirinae* subfamily, while Epstein-Barr virus (EBV) and Kaposi’s sarcoma-associated herpesvirus (KSHV) belong to the *Gammaherpesvirinae* subfamily (Foulon, 1992).

**FIGURE 1:**
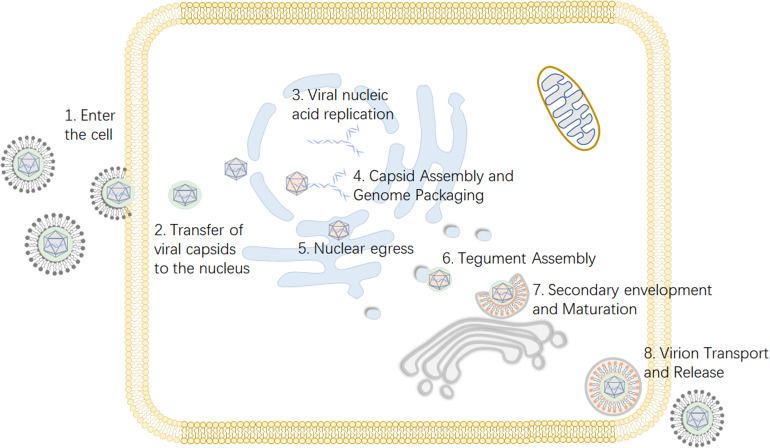
Viral replication process of alphaherpesviruses. **(1)** The virus enters the host cell through membrane fusion. **(2)** Viral proteins transport the viral nucleocapsid to the nuclear pore. **(3)** After the viral DNA enters the nucleus, the viral genome is transcribed, and replication begins. **(4)** The viral capsid is formed and subsequently packaged with nucleic acid. **(5)** Nuclear export, also known as the primary envelopment and de-envelopment, occurs. **(6)** The assembly of tegument on capsids occurs predominantly in the cytoplasm following nuclear egress. **(7)** Secondary envelopment, which enables the virus to reach maturity, occurs. **(8)** The mature virus is released from the cell. Some inspiration for this figure was obtained from these previous articles (Zeev-Ben-Mordehai et al., 2014; You et al., 2017).

Viruses depend on host cells for proliferation, so viruses enter host cells through endocytosis and membrane fusion (Mercer et al., 2010). When a viral particle infects a cell, viral envelope glycoproteins mediate the fusion of the viral envelope and the cell membrane (Johnson and Baines, 2011). Subsequently, the viral nucleocapsid is released into the cytoplasm, and viral DNA migrates into the nucleus through the nuclear pore and begins to replicate, initiating transcription of the viral genome (Liashkovich et al., 2011). The capsid is assembled in the nucleus, and then the genome is packed into the preformed capsid (Heming et al., 2017). Next, primary envelopment and de-envelopment occurs through budding into the inner nuclear membrane and subsequent fusion with the outer nuclear membrane (Bigalke and Heldwein, 2016). During secondary envelopment a subset of tegument proteins are incorporated into the viral particle (Kramer and Enquist, 2013). Virions are assembled in the lumena of large cytoplasmic vesicles that are transported to the cell periphery, where they fuse with the plasma membrane and release viral particles from the cell (Crump, 2018) (Figure 1).

Herpesvirus particles are approximately spherical, with sizes varying from 120 to 300 nm, and they are mainly composed of four parts from outside to inside: the envelope, tegument, capsid, and DNA genome core (Dargan, 1986; Roizman and Sears, 1996; Xie et al., 2019). The linear dsDNA genome is covered with an icosahedral capsid. The layer of protein between the capsid and outer envelope, collectively termed the tegument, is unique to herpesvirus (Metrick et al., 2020). Twenty-six tegument proteins have been found in HSV-1 (Loret et al., 2008; Kelly et al., 2009; Kukhanova et al., 2014). Tegument proteins play different roles during the viral life cycle, serving as structural components of the virion and functioning in transcriptional regulation, kinase activity, viral assembly, and immune evasion (Owen et al., 2015; Wang Y. et al., 2018; Tobler et al., 2019; Yang et al., 2019); for example, the Us3 protein can regulate the expression of viral genes, affect the immune function of the host, affect the spread of the virus between host cells, resist host cell apoptosis, and disrupt the host cell cytoskeleton (Van den Broeke et al., 2009; Chang et al., 2013; Grauwet et al., 2016).

The tegument protein VP22 possesses a protein transport function: it can carry a connecting fusion protein directly across the cell membrane into a cell without assistance, and the transported protein retains its original biological activity and function (Yu et al., 2012). In recent years, many studies have proposed the potential development and application of VP22 transduction in gene therapy (Ma et al., 2005; Nishi and Saigo, 2007; Yu X. et al., 2015; Yu et al., 2016), but the role of VP22 in the viral life cycle has not been fully elucidated. This article reviews the regulatory and structural role of alphaherpesvirus VP22 in the viral life cycle. According to the characteristics of VP22, we also propose some hypotheses and reflect on problems that remain to be solved.

## Features of VP22

VP22 is found only in alphaherpesviruses and not in beta or gammaherpesviruses (Kelly et al., 2009). VP22 is encoded by a single gene, which is most commonly (but not always) designated UL49 in alphaherpesviruses (Wu et al., 2012). For example, the homologous protein of VP22 found in VZV is ORF9p, which is encoded by the ORF9 gene, and ORF9 is the most transcribed VZV gene during viral infection (Kennedy et al., 2005).

It is estimated that nearly 2,000 copies of VP22 are present within each virion, and despite its abundance, very little is known regarding the role of VP22 during viral infection (Heine et al., 1974). VP22 has a predicted molecular weight of 32 kDa, which is below the size limit for passive diffusion through nuclear pores (molecular weight of 40–45 kDa) (Elliott and Meredith, 1992; Leslie et al., 1996). Deletion of the UL49 gene impacts viral replication differently in different virus species. For MDV, EHV-1, and VZV, VP22 (or ORF9) is indispensable for viral replication in cultured cells (Dorange et al., 2002; Che et al., 2008; Okada et al., 2015), but for BoHV-1, PRV, and HSV, VP22 is dispensable for viral replication in cultured cells (Liang et al., 1995; del Rio et al., 2002; Duffy et al., 2006). However, the absence of VP22 weakens pathogenicity in natural hosts infected with BoHV-1 and in animal infection models of HSV, impairing viral replication and altering the protein composition of assembled virions in infected cells (Pomeranz and Blaho, 2000; Elliott et al., 2005).

### Viral Gene Encoding VP22

The HSV-1 UL49 gene (NC_001806.2), which encodes VP22, is located in the UL48-UL49-UL49.5 region of the genome between the complementary sequences of the UL49.5 gene and the UL48 gene. The orientation of the UL49 gene is the same as t the UL48 but opposite to the UL49.5 gene (Elliott and Meredith, 1992). The length of the UL49 gene differs somewhat among different alphaherpesviruses and generally ranges from 600 to 1,000 bp (Table 1).

**TABLE 1:** Features of alphaherpesvirus UL49 gene and homologs.

Subfamily	Virus name	Abbreviation	Genomic sequence	Coding region	Coded protein	Number of bases
*Alphaherpesvirinae*	Herpes simplex virus 1	HSV-1	NC_001806.2	UL49	VP22	906
	Herpes simplex virus 2	HSV-2	NC_001798.2	UL49	VP22	903
	Varicella-zoster virus	VZV	NC_001348.1	ORF9	VP22	909
	Saimiriine alphaherpesvirus 1	SaHV-1	NC_014567.1	UL49	VP22	906
	Ateline alphaherpesvirus 1	HVA1	NC_034446.1	UL49	VP22	906
	Macacine alphaherpesvirus 1	CeHV-1	NC_004812.1	UL49	VP22	870
	Psittacid alphaherpesvirus 1	PsHV1	NC_005264.1	UL49	VP22	855
	Duck enteritis virus	DEV	NC_013036.1	UL49	VP22	762
	Pseudorabies virus (Suid alphaherpesvirus 1)	PRV (SuHV-1)	NC_006151.1	UL49	VP22	750
	Gallid alphaherpesvirus 3	MDV-2	NC_002577.1	UL49	VP22	726
	Bovine alphaherpesvirus 1	BoHV-1	NC_001847.1	UL49	VP22	777
	Felid alphaherpesvirus 1	FeHV-1	NC_013590.2	UL49	VP22	1038
	Testudinid herpesvirus 3	TeHV-3	NC_027916.2	UL49	VP22	639
	Meleagrid alphaherpesvirus 1	MeHV-1	NC_002641.1	HVT057	VP22	852
	Equid alphaherpesvirus 1	EHV-1	NC_001491.2	ORF11	VP22	915

According to the temporal sequence of gene expression during herpesvirus infection, viral genes are classified into immediate early (IE or α) genes, early (E or β) genes, and late (L or γ) genes. The late proteins are involved in virus assembly, maturation, and egress. The late genes are further subdivided into γ1 genes (which are partially dependent on viral DNA synthesis) and γ2 genes (which are highly dependent on viral DNA synthesis) (Gruffat et al., 2016). The UL49 gene belongs to the γ1 gene group and has been conserved during the evolution of alphaherpesviruses (Mouzakitis et al., 2005).

### Structure of VP22

#### Secondary Structure and Core Domain

HSV-1 VP22 is capable of forming higher-order protein structures consisting of dimers or tetramers (Taddeo et al., 2007). Sequence analyses and secondary structure predictions have revealed that VP22 consists of a non-conserved N-terminal domain and a conserved C-terminal domain with clear secondary structures (O’Regan et al., 2007a). The VP22 core domains of VZV, PRV, MDV, and infectious laryngotracheitis virus (ILTV) all have conserved secondary structures (3 alpha helices and 1 beta chain) (Drozdetskiy et al., 2015). X-ray crystallography has revealed that VP22 of HSV-1 shares extensive structural similarity with ORF52 of Murid herpesvirus-68 (MHV-68) (subfamily *Gammaherpesvirinae*) (Song et al., 2005; Bortz et al., 2007). Comparisons of the sequences of VP22 in different viruses have indicated that the core domain length is 78–79 amino acids and is present in different positions of VP22 homologs (Trapp-Fragnet et al., 2019).

#### Phosphorylated Protein

VP22 is a posttranslationally modified phosphoprotein (Knopf and Kaerner, 1980; Hutchinson et al., 2002). The protein responsible for its phosphorylation varies in different herpesviruses. For example, the CKII and HSV-1 UL13 genes encode protein kinases that are responsible for the phosphorylation of VP22 (Elliott et al., 1996; Asai et al., 2007). In VZV, a protein kinase encoded by the ORF47 gene is responsible (Riva et al., 2013). In addition, a protein kinase encoded by the US3 gene can weakly phosphorylate VP22 in BoHV-1 (Labiuk et al., 2010). However, VP22 exists in both phosphorylated and non-phosphorylated forms in infected cells, and only hypophosphorylated VP22 is incorporated into virions, and this dephosphorylation occurs independently of viral DNA replication or virion assembly (Pinard et al., 1987; Meredith et al., 1991; Geiss et al., 2001).

#### Nuclear Localization Signal (NLS) and Nuclear Export Signal (NES)

Although BoHV-1 VP22 is rich in basic amino acids, an analysis of the amino acid sequence of VP22 has shown that it does not have a classical nuclear localization signal (NLS) sequence; it accumulates in the nucleus of infected cells by an unknown mechanism (Zheng et al., 2005). Atypical nuclear localization signals refer to NLS without certain sequence characteristics. They are mainly present in proteins that can shuttle between the nucleus and cytoplasm. Transient transfection experiments with a fluorescently labeled fusion protein have further shown that the nuclear localization of VP22 is independent from that of other viral genes (Harms et al., 2000). In addition, many studies have reported that the C-terminus of BoHV-1 VP22 is essential for its nuclear localization (Ren et al., 2001; Zheng et al., 2005; Zhu et al., 2005). For example, one study has shown that amino acids 130–232 in BoHV-1 VP22 form the nuclear targeting sequence, which is capable of binding to histone H4, resulting in nuclear retention of VP22. In addition, a mitochondrial targeting sequence was present in the C-terminal 49 amino acids of VP22, overlapping the sequence required for nuclear targeting (Zhu et al., 2005). Other studies have demonstrated that the nuclear targeting sequence of BoHV-1 VP22 is located at amino acids 121–139, while amino acids 130–133 of VP22 form a functional non-classical NLS; amino acids 204–216 of VP22 have also been detected to form a nuclear export signal (NES) (Zheng et al., 2005). Further research has shown that amino acids 131–134 of BoHV-1 VP22 are not required for large-scale nuclear transport of proteins in later stages of infection but are essential for targeting of VP22 to discrete spot-like nuclear domains in the cytoplasm in the early stage (Lobanov et al., 2010). Overexpressed equine herpesvirus 1 (EHV-1) VP22 localizes to the cytoplasm and nucleus, and the phenomenon of VP22 transfer to the nucleus is more obvious under viral infection than under non-infection conditions; furthermore, amino acids 154–188 of EHV-1 VP22 have been confirmed to be the NLS via truncation experiments (Okada et al., 2014).

#### Functional Region

HSV-1 VP22 associates with cellular membranes and enables transmembrane transport via a functional domain located between residues 120 and 225. The association of VP22 with cellular membranes may prove instrumental for the proper tegumentation and envelopment of virions. Interestingly, the primary structural alignment has revealed that the region of HSV-1 VP22 within amino acids 120–225 is highly conserved among VP22 homologs of herpesvirus, which raises the possibility that membrane association is a conserved attribute of VP22 (Brignati et al., 2003). In one study, the recombinant virus HSV-1 (RF177) was found to produce a novel fusion protein between the last 15 amino acids of VP22 and GFP, termed GFP-VP22, which is integrated into the RF177 virus particles, suggesting that the information required for virus particle targeting is located in the last 15 amino acids of VP22 (Schlegel and Blaho, 2009). HSV-1(F) VP22 has two dileucine motifs, one at amino acids 235 and 236 and one at amino acids 251 and 252, and these motifs are required for the proper cytoplasmic localization of the virus within cells (Tanaka et al., 2012). The core region (amino acids 1–190) at the N-terminus of MDV VP22 is essential for virus spread, nuclear localization, histone association, and cell cycle regulation. However, amino acids 191–249 of VP22 are not essential for MDV transmission; rather, they facilitate efficient cell-to-cell spread. Amino acids 174–190 are essential for MDV VP22 functionality, and the IKIT motif (amino acids 159–162), located in the predicted β-strand, plays key roles in nuclear localization, histone association, and cell cycle arrest (Trapp-Fragnet et al., 2019) (Table 2).

**TABLE 2:** VP22 function and its functional domain.

Virus	Protein	Function	Functional domain	References
HSV-1	VP22	Associates with cellular membranes	120–225 amino acids	Brignati et al., 2003
		Virus particle targeting	The last 15 amino acids of VP22	Schlegel and Blaho, 2009
		Cytoplasmic localization	Two dileucine motifs (amino acids 235 and 236 and amino acids 251 and 252)	Tanaka et al., 2012
		Stabilize the microtubule network	Not studied	Elliott and O’Hare, 1998
		Inhibits CD1d recycling	Not studied	(Liu et al., 2016)
		Closely related to mitotic chromosomes	C-terminus	Ingvarsdottir and Blaho, 2010
		Associate directly with nucleoli	Not studied	López et al., 2008
MDV	VP22	Virus spread, nuclear localization, histone association, and cell cycle regulation	1–190 amino acids	Trapp-Fragnet et al., 2019
		Nuclear localization, histone association, and cell cycle arrest	The IKIT motif (amino acids 159–162)	Trapp-Fragnet et al., 2019
		Regulates the cell cycle	Not studied	Trapp-Fragnet et al., 2014
		Induces DNA double-strand breaks	C-terminus	Bencherit et al., 2017
BoHV-1	VP22	Promotes apoptosis of host tumor cells	Not studied	Qiu et al., 2005

#### Localization in Virions

During herpesvirus infection, the envelope fuses with the cell membrane to allow viral particles to enter a cell. Most of the tegument and the envelope detach from the virus in the cytoplasm, releasing the nucleocapsid and some tegument proteins (Maurer et al., 2008). The tegument proteins of herpesviruses are generally divided into “inner” and “outer” tegument proteins. The inner tegument proteins are closely related to the nucleocapsid, while the outer membrane proteins are weakly related to the inner surface of the nucleocapsid and envelope. During PRV infection, the inner tegument proteins (added first during assembly), including pUL36, pUL37, and pUS3, remain associated with the capsid during viral entry, while outer tegument proteins such as pUL11, pUL47, pUL48, and pUL49 are lost (Granzow et al., 2005). Because VP22 interacts with the cytoplasmic tail of the glycoprotein at the Golgi apparatus to participate in secondary envelopment, VP22 is mainly located in the outer tegument close to the envelope in virions.

### VP22 Subcellular Localization

The distribution of the VP22 in cells is dynamic. For example, during HSV-1 infection, the protein exhibits at least three different subcellular distributions: cytoplasm only, cytoplasm and nucleus, and nucleus only. VP22 is mainly distributed in the cytoplasm in the early stages of viral infection, but it migrates to the nucleus in the late stage of infection, and nuclear VP22 is more highly phosphorylated than cytoplasmic VP22 (Pomeranz and Blaho, 1999; Sugimoto et al., 2008). VP22 exhibits a diffuse distribution in the cytoplasm and nucleus in MDV-infected cells and accumulates in the nucleus when MDV-VP22 is overexpressed (Dorange et al., 2000). HSV-1 VP22 and BoHV-1 VP22 are known to localize in the cytoplasm and the nucleus independently of other viral proteins (Harms et al., 2000; Blouin and Blaho, 2001).

One study found that VP22 is localized in the cytoplasm before microtubule rearrangement and in the nucleus after microtubule rearrangement. Drugs that stabilize microtubules can increase the accumulation of VP22 in the cytoplasm, and VP22 colocalizes with microtubules. Thus, it was concluded that the microtubule reorganization during HSV-1 infection promoted the nuclear localization of VP22. During HSV-1 infection, microtubule interaction may present a means by which VP22 avoids nuclear localization during the early phase of the replication cycle (Kotsakis et al., 2001). During non-viral infection conditions *in vitro*, HSV-1 VP22 is mainly localized in the cytoplasm; however, during mitosis, VP22 begins to associate with chromatin and becomes relatively fixed in one position after entering the nucleus. In live cell analysis experiments, VP22 protein has been found to be mainly located in acidic cell regions and distributed in puncta that move quickly through the cytoplasm. This punctate distribution may be related to vesicle transport in the secretory pathway and the trans-Golgi network (TGN) (Elliott and O’Hare, 1999; Brignati et al., 2003). VZV ORF9p is present in the TGN and plays a role in the secondary envelopment process (Che et al., 2013; Riva et al., 2013).

## Role of VP22 in the Viral Life Cycle

### Effect of VP22 on Viral Gene Transcription

During herpesvirus infection, the virus cooperates with cell transcriptional regulatory factors to synergistically regulate transcription initiation. Notably, the expression of the ICP4 gene is significantly lower in cells infected with VP22-knockout EHV-1 than in those infected with the parent virus (Okada et al., 2018).

The product of the HSV-1 UL41 gene, Virion Host Shutoff (VHS) protein, is an endoribonuclease that degrades mRNA during the early stages of infection and inhibits host protein synthesis (Su and Zheng, 2017; He et al., 2020). Earlier, a study found that vhs protein binds VP22 only in the presence of VP16 to form a VP22-VP16-vhs tripartite complex that promotes the translation of viral mRNA, but it does not block the degradation of mRNA by vhs. The presence or absence of VP16 or VP22 does not affect the stable state of VHS mRNA, while the presence of VP16 and VP22 can enhance expression of VHS protein for assembly into virions (Taddeo et al., 2007). Recently, research has reported that during infection, translation of vhs requires VP22 but not the VP22-VP16 complex, and coexpression of VP16 and VP22 can rescue the cytoplasmic localization of vhs mRNA but has failed to rescue vhs translation. The VP22-VP16-vhs complex uses nuclear retention of vhs mRNA instead of translation as a rescue target (Elliott et al., 2018).

Other studies found that in the absence of VP22, not only are early and late transcripts retained in the nucleus through VHS-dependent mechanisms, but this feature extends to cell transcripts that are not effectively degraded by VHS. Moreover, it reveals the true role of VP22 in the regulation of VHS activity, which specifically rescues the cytoplasmic localization of L transcripts rather than their hyper-degradation (Pheasant et al., 2018). Deletion of the HSV-1 UL49 gene generates three inactive VHS mutants: two lacking codons 22–75 of the UL41 gene and another with a codon frameshift at site 286. All the mutations cause vhs to be inactive in the absence of VP22 (Sciortino et al., 2007). In addition, deletion of the HSV-1 UL49 gene slightly decreases viral mRNA levels and causes defects in polysome assembly that are independent of mRNA abundance, but these defects can be rescued by a secondary compensatory frameshift mutation in vhs, indicating the existence of functional interplay between VP22 and vhs with regard to both accumulation and translation of viral mRNA (Mbong et al., 2012). The opposite is a study showing a replication-competent VP22 deletion mutant virus constructed by homologous recombination carrying a wild-type (Wt) vhs gene and no other gross mutations. Therefore, the mode of virus rescue affects the acquisition of secondary mutations (Ebert et al., 2013).

### VP22 Affects Viral Protein Synthesis

VP22 affects viral gene expression and protein synthesis. Deletion of HSV-1 VP22-encoding gene (UL49) can lead to modest reductions in levels of glycoproteins gD and gB. Experimental data indicate that VP22 is involved in recruiting ICP0 (and potentially ICP4) to specific cytoplasmic domains and is necessary to assemble the immediate early proteins ICP0 and ICP4 between the envelopes of HSV particles. In addition, VP22 influences both the intracellular levels and localization of ICP0 (Elliott et al., 2005). Further research has shown that VP22 enhances the accumulation of gE and gD mRNA in the early stage of HSV-1 infection but has no effect in the late stage of infection; however, deletion of VP22 causes the synthesis of most proteins to cease in the late stage of infection, and this decline in protein synthesis is not related to abnormal apoptosis or increased eIF-2a phosphorylation. Overall, VP22 promotes viral mRNA accumulation in early stages and protein synthesis in late stages of infection (Duffy et al., 2009).

HSV-1 VP22 can interact with the TATA box binding protein-associated factor (TAF-I) protein to inhibit the assembly of nucleosomes on DNA, but it does not affect the inhibitory activity of the histone acetyltransferase inhibitor INHAT on the histone acetyltransferase P300 or PCAF. TAF-I can also block the non-specific binding of VP22 to DNA. Since overexpression of TAF-I interferes with the process of HSV-1 infection in transfected cells, researchers have speculated that VP22 may interfere with TAF-I-mediated ribosomal binding to mRNA in the early stage of HSV infection (van Leeuwen et al., 2003). This phenomenon suggests that VP22 may promote the expression of viral genes by maintaining important regulatory sequences in viral gene promoters in a nucleosome-free state so that they can be accessed by transcription factors.

In one study, a VP22 251 and 252 dileucine motif mutant and a VP22 restoration virus of the wild-type HSV-1 strain HSV-1(F) (viruses: VP22LL251AA and VP22LL251AA-repair, respectively) were inoculated into the brains of mice. The 50% lethal dose (LD50) of VP22LL251AA in the brain was approximately 10^3^-fold lower than VP22LL251AA-repair at 3 and 5 days after infection. In addition, viral antigens were detected in many regions of the brain in VP22LL251AA-repair-infected mice but only in restricted regions of the brain in VP22LL251AA-infected mice, confirming that VP22 is a significant neurovirulence factor *in vivo* (Tanaka et al., 2012).

### VP22 Influences Virion Assembly

#### Interactions With Viral Tegument Proteins

To date, research examining how VP22 is assembled into virions has revealed inconsistent results. Early studies have suggested that an 89-amino acid sequence at the C-terminus of HSV-1 VP22 is sufficient for packaging a small amount of VP22 into virions, but VP22 must interact with VP16 to be packaged into virions at wild-type levels, indicating that VP16 is important for this process (Hafezi et al., 2005). In contrast, a study assessing the effect of HSV-1 VP22 binding with VP16 or gE on VP22 packaging into virions and related compartmentalization patterns later revealed that VP22 does not need to interact with VP16 to be packaged into virions (O’Regan et al., 2007b).

VZV ORF9p can interact with amino acids 1–43 of the IE62 acidic transactivation domains (TADs) and with tubulin, suggesting a model for ORF9p function in which ORF9p forms a complex with IE62 and possibly other tegument proteins in the cytoplasm at late stages of infection (Cilloniz et al., 2007). HSV VP22 can directly interact with the TAD in the C-terminus of VP16. When VP22 and VP16 are coexpressed in the absence of other viral proteins, they colocalize around the nuclear membrane, which may reflect their functions in viral assembly (Elliott et al., 1995). VZV ORF9p interacts with amino acids 549–793 at the C-terminus of ORF11p, and both proteins are located mainly in the *trans*-Golgi vesicle region in infected cells. This region participates in the secondary envelopment of viral particles, suggesting that the ORF9 protein plays key roles in VZV particle membrane assembly and secondary envelopment (Che et al., 2013).

Deletion of PRV pUL21 reduces the amounts of pUL46, pUL49 and pUS3 packaged into virions by 80–90%, indicating that pUL21 is very important for pUL49 packaging into virions (Michael et al., 2007). The HSV-1 UL16 gene encodes a protein that can interact with VP22, and deletion of the UL16 gene can disrupt the participation of VP22 in viral packaging. Deletion of VP22 reduces the packaging of gE and VP16 but not the packaging or expression of the UL16 gene, which confirms that the assembly of VP22 into virions depends on UL16 (Starkey et al., 2014).

Earlier studies found that ICP0 could be easily detected in wild-type (Wt) virus particles, but ICP0 could not be detected in virus preparations lacking the major tegument protein VP22, suggesting that VP22 is somehow involved in the assembly of ICP0 (Elliott et al., 2005). Furthermore, as VP22 is required for assembly of the virion of the immediate early protein ICP0, any interaction that affects the recruitment of VP22 to viral particles may indirectly affect the assembly of ICP0 (Maringer and Elliott, 2010).

VZV ORF9p with a point mutation has been found to be able to interact with and be phosphorylated by the CKII-like serine/threonine kinase ORF47p during infection (Oral et al., 2016), but mutation of the amino acid E85 in ORF9p severely affects the assembly and release of the virus. Therefore, the phosphorylation of amino acid E85 in ORF9p is essential for primary envelopment of VZV virions and nucleation of virions during viral transmission (Riva et al., 2013).

#### Interactions With Viral Glycoproteins

Entry of most, but not all, herpesviruses into cells requires the envelope glycoprotein gD (Pannhorst et al., 2018). HSV VP22 can specifically bind to the cytoplasmic tail of gD, and this binding depends on the arginine and lysine residues at positions 5 and 6 of the gD cytoplasmic tail. In addition, the HSV-1 capsid binds to the cytoplasmic tail of gD and exhibits a similar sequence dependence. Therefore, VP22 can act as a linker protein to mediate the interaction of the HSV capsid with gD (Chi et al., 2005). Subsequently, a team confirmed by reciprocal pull-down experiments that HSV-1 VP22 interacts with gD in a specific manner, and these interactions require the CT domain of gD (Farnsworth et al., 2007). Although gD has been described as a VP22 binding partner, one study has not been able to repeat the interaction between VP22-gD (Maringer et al., 2012).

A study revealed that a mutant form of HSV-1 lacking 14 residues of VP22 (and thus unable to interact with gE) does not grow well in epithelial cells; in fact, the growth phenotype was the same as that observed after deletion of the complete VP22 ORF, suggesting that gE is essential for packaging of VP22 into virions (Stylianou et al., 2009). To further confirm the role of HSV-1 VP22/gE binding in promoting viral particle packaging, a team used site-directed mutagenesis to specifically destroy the binding activity of VP22 and gE, and their findings confirmed that VP22 is packaged into virions independently of any interaction with gE (O’Regan et al., 2010). HSV-1 VP22 has also been reported to interact with the glycoproteins gE and gM (O’Regan et al., 2007a; Stylianou et al., 2009). The glycoprotein-binding domain of VP22 is conserved among all VP22 homologs, suggesting that the gE-VP22-gM complex may be important for alphaherpesvirus replication (Mouzakitis et al., 2005).

In addition, studies have demonstrated that the C-terminus of PRV VP22 binds to the cytoplasmic tails of gE and gM. Deletion of VP22 does not affect viral maturation or secondary envelopment in the cytoplasm. Under normal infection conditions, VP22 is effectively packed into virions; however, the absence of the gE-gl complex and gM affects secondary envelopment and prevents VP22 from being packaged into virions (Fuchs et al., 2002). BoHV-1 VP22 can interact with the glycoprotein gN encoded by the UL49.5 gene without relying on gM. In the absence of gM, the assembly of gN into the viral envelope may be mediated by an interaction of amino acids 80–96 in the intracellular domain of gN with VP22 (Pannhorst et al., 2018).

The herpesvirus tegument exerts the roles of other viral matrix proteins by interacting with the capsid on one side and the cytoplasmic tails of envelope glycoproteins on the other side, connecting these structural components for the final envelopment process and ensuring the integrity of the virus particle (Klupp et al., 2017). The envelope formation and scission involve the participation of multiple viral proteins and cellular ESCRT devices. For HSV-1 and PRV, a number of tegument and envelope proteins are assigned roles in the envelopment, and may provide a pathway for the recruitment of ESCRT complexes, which contains five tegument proteins (encoded by HSV-1 UL21, UL46, UL47, UL48, and UL49) (Owen et al., 2015; Barnes and Wilson, 2019).

**FIGURE 2:**
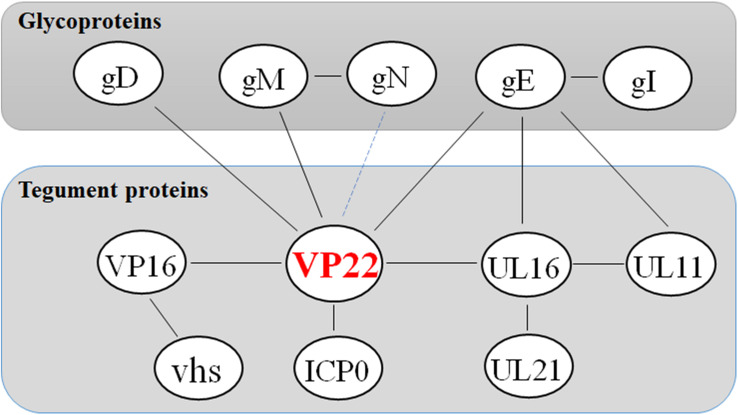
Network of protein–protein interaction around the tegument protein VP22. Some inspiration for this figure was obtained from a previous article (Maringer et al., 2012).

Studies have shown that VP22, ICP0, gE, and gM are present within the same complex in infected cells, and that although VP22 is not required for virus assembly, it is fundamental to the formation of this multicomponent complex (Maringer et al., 2012). This finding reveals the existence of specific interactions between herpesvirus the cytoplasmic tails of envelope glycoproteins and tegument protein VP22. These interactions may facilitate the secondary envelope of the herpesvirus viral particles during maturation, thereby ensuring the integrity of the virus particles (Figure 2 and Table 3).

**TABLE 3:** Tegument protein VP22 and its interacting virus protein.

Virus	Protein	Interacting Virus protein	Function	References
HSV-1	VP22	gE	Packaged into virions	Stylianou et al., 2009
		gM	VP22-gM association may be dynamic, packaged into virions	Mouzakitis et al., 2005
		gD	Mediates the interaction of the HSV-1 capsid with gD	Chi et al., 2005
		ICP0	VP22 is required for virion assembly of ICP0	Maringer and Elliott, 2010
		VP16	This interaction is not required to package VP22 into virion;	O’Regan et al., 2007b
		UL16	Packaged VP22 into virions	Starkey et al., 2014
PRV	VP22	gE	Packaged into virions	Fuchs et al., 2002
		gM	Packaged into virions	Fuchs et al., 2002
BoHV-1	VP22	gN	In the absence of gM, the assembly of gN into viral membrane	Pannhorst et al., 2018
VZV	ORF9p	IE62	Forms a complex with other tegument proteins in the cytoplasm at late stages of infection	Cilloniz et al., 2007
		ORF11p	VZV particle membrane assembly and secondary envelopment	Che et al., 2013
		ORF47p	The primary envelopment of VZV virions and the nucleation of virions	Oral et al., 2016

#### Interactions With Cellular Protein

VZV ORF9p can interact with cell adapter protein complex 1 (AP-1), and leucine 231 in ORF9p has been identified as critical for this interaction. Disruption of ORF9p binding to AP-1 via point mutation of ORF9p severely impairs viral growth, most likely by preventing effective viral secondary envelopment (Lebrun et al., 2018). MDV VP22 and HSV-2 VP22 also interact with AP-1 (Qiu et al., 2005). In HSV-1 infected cells, VP22 may be activated via phosphorylation mediated by protein kinases encoded by the CKII and UL13 genes (Elliott et al., 1996; Asai et al., 2007). In a study in which a high multiplicity of infection (MOI) and actinobacteria were used to artificially increase the amount of virus in infected cells and stop the infection process, the tegument protein VP22 of HSV-1 was found to be phosphorylated upon viral entry into cells, which mediated VP22 dissociation from the virion *in vitro*. With the UL13 mutant virus, the release of VP22 was significantly impaired, suggesting that the protein kinase encoded by the UL13 gene is a major protein kinase facilitating the separation of VP22 from virions (Morrison et al., 1998) (Table 4).

**TABLE 4:** Tegument protein VP22 and its interacting cellular protein.

Virus	Protein	Interacting cellular protein	Function	References
HSV-1	VP22	TAF-1	Inhibits the assembly of nucleosomes on DNA	van Leeuwen et al., 2003
		NMIIA	Lined up with filaments containing NMI IA	van Leeuwen et al., 2002
		cGAS	Inhibits the enzymatic activity of cGAS	Huang et al., 2018
		AIM2	Prevents AIM2 oligomerization	Maruzuru et al., 2018
BoHV-1	VP22	Histone H4	Reducing H4 histone acetylation	Ren et al., 2001
VZV	ORF9p	AP-1	Effective viral secondary envelopment	Lebrun et al., 2018

### VP22 Promotes Cell-to-Cell Viral Transmission

Many viruses can trigger remodeling of the host cytoskeleton in their early life cycle to promote efficient entry into the host cell and transport to the nucleus (Zandi et al., 2013; Cirillo et al., 2017; Dharan and Campbell, 2018). HSV-1 VP22 is transiently expressed in cells, colocalizes with a unique cytoplasmic filamentous structure, and can induce microtubule recombination into a thick bundle structure. Analysis of the infected cells further showed that VP22 co-localized with microtubules during the infection and induced microtubule acetylation. Moreover, the resistance of VP22-induced microtubule bundles to microtubule-depolymerizing agents and cold treatment was stronger than that of normal cell microtubules, suggesting that VP22 could stabilize the microtubule network (Elliott and O’Hare, 1998).

PRVs lacking tegument proteins such as VP13/14, VP16, and VP22 can enter cells but cannot translocate normally to the nucleus (Mettenleiter, 2002). A team investigated the protein profile of VP22-null (PRV 175) virions for differences relative to wild-type virions, the results show that in the absence of VP22, tegument assembly compensates in a limited manner by increasing incorporation of cellular actin (del Rio et al., 2005). The structures and functions of the core domains of the HSV-1 VP22 and the MHV-68 ORF52 protein are very similar, indicating that both proteins can serve as adaptors to bind different proteins. VP22 and ORF52 can interact with many proteins and regulate protein localization in cells to participate in the assembly of a protein scaffold consisting of other tegument proteins and to establish a protein bridge between the capsid and the lipid envelope. This assembly process may be important for intracellular transport of proteins along microtubules (Hew et al., 2015).

Myosin II present in non-muscle cells is called non-muscle myosin II (NMIIA). In addition to serving as a molecular motor for various molecular movements in cells, NMIIA participates in cell migration, adhesion, cytokinesis, vesicle secretion, Golgi budding, and other physiological activities (Vicente-Manzanares et al., 2009; Arii et al., 2010). A study found that viral particles of an HSV-1 recombinant virus expressing the fusion protein GFP-VP22 could be observed that colocalized with a population of NMIIA in sites proposed to be viral assembly compartments in the late stage of infection. In addition, GST-pulldown experiments of cell samples and prokaryotic samples demonstrated that VP22 could interact with NMIIA, but it remained formally possible that the NMIIA-VP22 association was indirect via NMIIA interactions with one of the other bound species (van Leeuwen et al., 2002).

Apoptosis in the early stage of viral infection prevents replication of the virus, but apoptosis in the late stage of infection is beneficial for the release and spread of progeny virus (Oral et al., 2016; Zhou et al., 2017). Transient expression of BoHV-1 VP22 in NXS2 cells does not affect the cell cycle, and analyses of caspase-3 activity and apoptosis have suggested that VP22 induces apoptosis in host tumor cells by upregulating the expression ratio of Bax to Bcl-2 (Qiu et al., 2005). These results suggest that VP22 may promote the spread of viruses by stimulating apoptosis in the late stage of viral infection.

The proteins encoded by the HSV-1 UL47, UL49, and US11 genes can bind to RNA in host cells and then be packaged into viral particles that are capable of carrying the proteins to newly infected cells for expression, thereby creating an environment that is conducive to effective triggering of infection (Sciortino et al., 2002). A study constructed the HSV-1 UL49 deletion and reverted strain viruses and analyzed their growth in cultured cells and mouse cornea compared with the parental strains. VP22 is dispensable for virus replication at high multiplicities of infection (MOIs), and analyses of plaque morphology and intra- and extracellular multistep growth identified a role for VP22 in viral spread during HSV-1 infection at low MOIs. In addition, viral spread in the mouse cornea was significantly reduced upon infection with the UL49-null virus compared with infection with the wild-type and UL49-repaired viruses, identifying a role for VP22 in viral spread *in vivo* and *in vitro* (Duffy et al., 2006). A team has constructed different BoHV-1 mutants with deletions of UL49 or gE, or both, to investigate the function of BoHV-1 VP22 in viral cell-to-cell spread. Deletion of gE resulted in a 53% reduction in plaque diameter, while a single deletion of UL49 resulted in a 52% reduction. However, the simultaneous deletion of gE and UL49 resulted in a 83% reduction in the diameter of the virus plaque, indicating that gE and VP22 are two equally important factors for BoHV-1 cell-to-cell spread and that both are likely to act independently of each other in a critical pathway of virus cell-to-cell spread (Kalthoff et al., 2008).

### VP22 Facilitates the Viral Life Cycle Through Innate Immune Evasion

**FIGURE 3:**
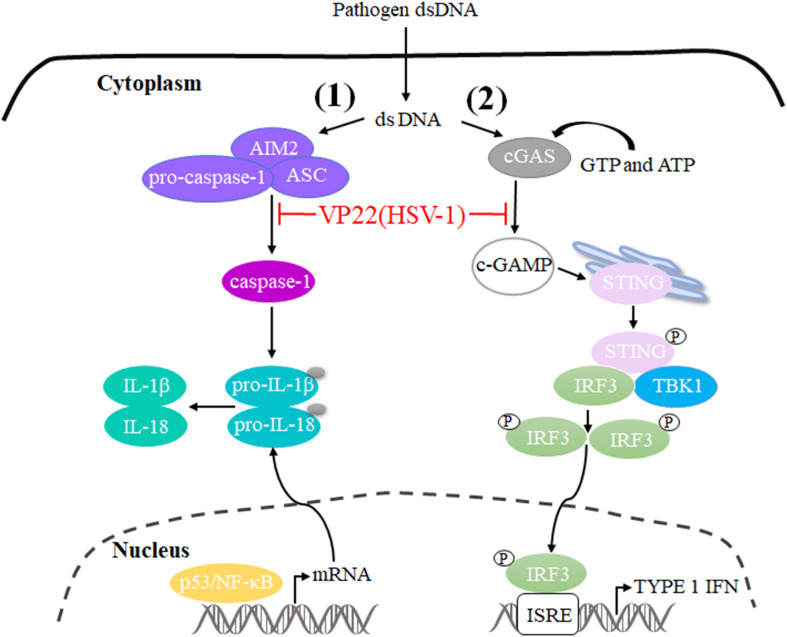
Schematic diagram of pathogen-derived molecules enabling VP22 to evade the DNA-sensing pathway in cells. **(1)** cGAS is activated after it binds to dsDNA and uses ATP and GTP to generate cGAMP through enzymatic activity. cGAS is responsible for activation of the binding protein STING. Phosphorylated STING begins to recruit TBK1 to activate IRF3, and phosphorylated IRF3 enters the nucleus to activate the interferon-stimulated response element (ISRE), thereby producing type I interferon to fight pathogens. HSV-1 VP22 binds to and inhibits the enzymatic activity of cGAS, thereby enabling evasion of the host’s innate immune response. **(2)** AIM2 is a pattern recognition receptor that can recognize dsDNA in the cytoplasm and bind to the linker protein ASC and pro-caspase-1 to form the AIM2 inflammasome (AIM2-ASC-Pro-caspase-1), which activates caspase-1; activated caspase-1 can cleave pro-IL-1β and pro-IL-18, which leads to the maturation and secretion of the inflammatory factors IL-1β and IL-18. HSV-1 VP22 can interact with AIM2 and prevent its oligomerization, thereby inhibiting the activation of AIM2-dependent inflammatory bodies.

The innate immune system is the first line of host defense against invading pathogens. It is also the basis for adaptive immune activation and plays an important role in the elimination of viruses from hosts. Under the long-term selective pressure of host immune activity, viruses have developed a variety of mechanisms to antagonize the antiviral ability of the host and thereby promote viral infection and replication (Sun et al., 2013; Orzalli et al., 2015; Chiang et al., 2018). Cyclic GMP-AMP (cGAMP) levels decrease during HSV-1 infection, indicating that there is at least one viral component capable of inhibiting cGAMP synthase (cGAS) activity. Subsequently, VP22 directly binds to and inhibits cGAS enzymatic activity, leading to the downregulation of IFN-β production and thereby inhibiting cGAS/STING-mediated natural antiviral immune pathways (Huang et al., 2018). In addition, a study constructed a mutant virus lacking VP22 (HSV-1 ΔVP22), which activates and induces AIM2-dependent secretion of IL-1β and IL-18. They found that VP22 could interact with AIM2 and prevent its oligomerization, thereby inhibiting activation of the AIM2-dependent inflammasome. HSV-1 ΔVP22 infection results in diminished viral yields *in vivo*, but HSV-1 ΔVP22 replication is largely restored in AIM2-deficient mice, revealing that HSV-1 evades host immune responses to enable efficient viral replication *in vivo* (Maruzuru et al., 2018) (Figure 3).

Many herpesvirus viral proteins can evade the innate immune response of the host (Christensen et al., 2016; Liu et al., 2018; Yang et al., 2019; You et al., 2019). In addition to playing an important role in innate immune evasion, VP22 can also play a role in specific immune evasion (Liu et al., 2016). A previous study on HSV-1 infection has revealed that compared with wild-type strains, mutant strains lacking VP22 have impaired ability to inhibit CD1d-mediated antigen presentation. This phenomenon can be reversed after VP22 is restored, indicating that VP22 inhibits CD1d recycling (Liu et al., 2016).

### Other Functions of VP22

#### Chromatin Binding

Chromatin, a material with a linear structure composed of DNA, histones, non-histone proteins, and a small amount of RNA, is found in interphase nuclei and contains the genetic material of interphase cells (Clapier and Cairns, 2009). Previous studies have shown that the molecular weights of HSV VP22 and histones are very similar during gel electrophoresis and that the C-terminal sequence of VP22 and histone folding domain sequence are very similar. Therefore, VP22 may localize in the nucleus in a manner similar to that of histones and be closely related to mitotic chromosomes (Elliott and O’Hare, 2000; López et al., 2008; Ingvarsdottir and Blaho, 2010).

To investigate the relationship between HSV-1 VP22 and nucleoli, one study detected the localization of nucleolar proteins and viral proteins during viral infection and found that the VP22 present in VP22-expressing Vero cells surrounded nucleoli; in addition, a portion of the VP22 localized to areas of altered nucleoli and to marginalized chromatin in nuclei at 4 and 6 hours post-infection (hpi). These findings indicate that VP22 might associate directly with nucleoli and that nuclear VP22 targets unique subnuclear structures early during HSV-1 infection (<6 hpi) (López et al., 2008). Given that VP22 closely associates with chromosomes during mitosis and is located in the marginalized chromatin region, it may affect chromosomes by interacting with DNA-binding proteins.

Histone modification plays important roles in chromatin recombination, cell cycle control, and gene regulation. In BoHV-1-infected or VP22-expressing cells, VP22 can bind to histones and nucleosomes, thus reducing H4 histone acetylation. Since VP22 and histones have similar epitopes, it has been speculated that BoHV-1 VP22 may play a regulatory role in the replication process of TAF-1. MDV VP22 has the same function as BoHV-1 VP22 during interactions with histones (Trapp-Fragnet et al., 2014). Interestingly, a positive correlation between cell cycle modulation and VP22 histone association, but no relationship with MDV cell-to-cell spread function, has been observed (Trapp-Fragnet et al., 2019).

#### Regulation of the Cell Cycle

**FIGURE 4:**
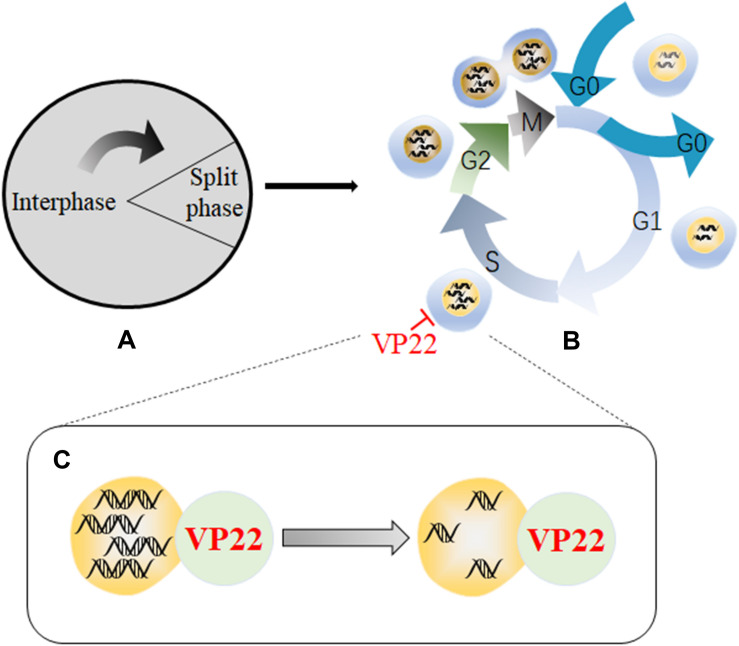
Schematic diagram of the mechanism by which VP22 induces cell cycle arrest. **(A)** The cell cycle is divided into an interphase and a cell division phase. **(B)** The interphase and cell division phase together include a resting phase (G0), an early stage of DNA synthesis (G1), a period of DNA synthesis (S), a late stage of DNA synthesis (G2), and a mitotic stage (M). **(C)** Through interactions with DNA, VP22 can cause a large number of DNA double-strand breaks to form, resulting in arrest in the S phase of the cell cycle, which is conducive to viral replication. Some inspiration for this figure was obtained from these articles (Mohni et al., 2013; Trapp-Fragnet et al., 2014).

Viral proteins can impede the life cycles of host cells through multiple cellular regulatory pathways that affect cell growth, and viruses use the functional proteins synthesized during the process to create the most suitable environment for viral replication (Liang et al., 2002; Yu J. et al., 2015; Wang Z. et al., 2018). Studies have shown that a recombinant MDV expressing a VP22 with a C-terminal GFP tag is highly attenuated effect *in vivo*, indicating that VP22 plays a role in MDV-induced tumorigenesis (Jarosinski et al., 2012). In addition, another study has found that MDV infection can arrest cells in S phase of the cell cycle, thereby disrupting cell cycle progression and slowing cell proliferation. In addition, 90% of cells that transiently express VP22 undergo cell cycle changes, indicating that VP22 is a major cell cycle regulator encoded by MDV (Trapp-Fragnet et al., 2014). Cell cycle arrest has been shown to be beneficial for herpesvirus replication, and overexpression of MDV VP22 can lead to cell cycle arrest in HSV-1- and VZV-infected cells (Flemington, 2001). Cell cycle arrest is related to DNA damage signals, which are usually triggered during viral infection, and the DNA damage response pathway is a priority target for herpesviruses (Mohni et al., 2013). A recombinant virus lacking the ability to induce DNA damage has showed defects in inducing tumors, suggesting that DNA damage induction may be involved in the tumorigenicity of MDV. Modification of the C-terminus of MDV VP22 disrupts the ability of VP22 to trigger DNA damage. Therefore, the VP22 C-terminus is essential for the induction of DNA damage, and VP22 is a major viral determinant associated with DNA damage (Bencherit et al., 2017). Therefore, the arrest in S phase induced by VP22 may produce a large number of DNA double-strand breaks (DABs) (Figure 4).

## Conclusion

The VP22 protein, a structural protein in alphaherpesviruses, is one of the most abundant tegument proteins, with more than 2000 copies in each virion (Heine et al., 1974). The replication process of herpesviruses includes adsorption, penetration, shelling, biosynthesis, virion assembly, maturation, and release. This virion assembly is a virus-specific process and can be used as an antiviral drug target. VP22 can interact with many tegument proteins and glycoproteins to regulate the assembly of viral particles, thereby mediating the movement of capsid proteins from the outer nuclear membrane of the cell to the Golgi apparatus and promoting the formation of mature viral particles, we suspect that VP22 can be used as a target for future research on antiviral drugs.

During the viral life cycle, VP22 participates in virion assembly, regulates the cell cycle, and functions in the transcriptional regulation of viral genes. VP22 can induce a large number of DNA double-strand breaks, and subsequent DDRs are beneficial for MDV replication, which is conducive to oncogenicity of MDV (Bencherit et al., 2017). In addition, VP22 can induce apoptosis of host tumor cells and thus facilitate the spread of the virus. These findings reveal a new mechanism of viral spread between cells and provide novel ideas and directions for further research on the treatment of diseases caused by herpesvirus infection.

In the early years, the literature has reported that proteins larger than −40 kda require an exposed NLS to enter the nucleus. The predicted molecular weight of HSV-1 VP22 is 32 kDa, which is below the size limit for passive diffusion through nuclear pores. However, not all proteins that fit the size of the protein can shuttle freely. Some proteins have a relatively small molecular weight but a special amino acid signal sequence, so they can enter the nucleus through active transport; or there is no signal sequence itself, but it can be associated with other signals, resulting in the entry of the combined material into the nucleus through active transport (Timney et al., 2016), then VP22 may need a special amino acid signal sequence to enter the nucleus through active transport.

Successful immune escape is the main factor underlying chronic herpesvirus infection. In recent years, there have been reports in the literature that VP22 participates in innate immune evasion. VP22 interacts with RNA and functions in the transport of viral mRNA, and packaged RNA can be expressed in newly infected cells (Sciortino et al., 2002). We further speculate that VP22 may inhibit innate immunity by transporting mRNA molecules that are translated into viral proteins in adjacent cells to suppress immune responses before the pattern recognition receptor pathway can recognize the newly infecting virus. By introducing specific mRNA into target cells, VP22 may prepare cells for the arrival of herpesvirus. However, further investigations are needed in the future to elucidate the details of this mechanism.

Further elaboration of the function and mechanism of VP22 will provide important reference data on the life cycles of herpesviruses in host cells and the interactions between pathogens and host cell molecules.

## Author Contributions

LP and AC contributed to the design of the manuscript. SM, XO, QY, YW, RJ ML, DZ, SC, and QG provided ideas contributing to the conception of this manuscript. BT helped to create the figures. MW modified the manuscript. All the authors read and approved the final manuscript for publication.

## Conflict of Interest

The authors declare that the research was conducted in the absence of any commercial or financial relationships that could be construed as a potential conflict of interest.
